# 2372. Preliminary evidence of durable immune responses induced by self-amplifying mRNA (samRNA) vaccine candidates against SARS-CoV-2 in vaccine-naive South African population

**DOI:** 10.1093/ofid/ofad500.1993

**Published:** 2023-11-27

**Authors:** A Koen, E Mitha, J Allagappen, A Nagare, M Dhar, M Marrali, Christine Palmer, Harshni Venkatraman, Jason Jaroslavsky, J C Kuan, L Kraemer, L Arcebuche, L Hernandez, Meghan Hart, Sonia Kounlavouth, Pedro Garbes, A Allen, Karin Jooss, Shabhir A Mahdi

**Affiliations:** Wits Vaccines & Infectious Diseases Analytics (VIDA) Research Unit, South Africa – Johannesburg (South Africa), Johannesburg, Gauteng, South Africa; Newtown Clinical Research Centre, South Africa - Newtown (South Africa), Newtown, Gauteng, South Africa; Setshaba Research Centre - Pretoria (South Africa), Pretoria, Gauteng, South Africa; Gritstone bio, Inc, San Jose, California; Shandukani Research - Johannesburg (South Africa), Johannesburg, Gauteng, South Africa; Gritstone bio, Inc, San Jose, California; Gritstone Bio, Cambridge, Massachusetts; Gritstone Bio, Cambridge, Massachusetts; Gritstone Bio, Cambridge, Massachusetts; Gritstone Bio, Cambridge, Massachusetts; Gritstone bio, Inc, San Jose, California; Gritstone bio, Inc, San Jose, California; Gritstone bio, Inc, San Jose, California; Gritstone Bio, Cambridge, Massachusetts; Gritstone Bio, Cambridge, Massachusetts; Gritstone bio, Inc., Weston, Massachusetts; Gritstone bio, Inc, San Jose, California; Gritstone bio, Inc., Weston, Massachusetts; University of the Witwatersrand, Johannesburg, South Africa, Johannesburg, Gauteng, South Africa

## Abstract

**Background:**

CORAL-CEPI (NCT05435027) is the first test of samRNA-based SARS-CoV-2 vaccines in the African population, under-represented in SARS-CoV-2 vaccine studies. Antibody transience is an issue with first generation SARS-CoV-2 vaccines. This is a preliminary safety and immunogenicity report, showing durable IgG and neutralizing antibody (nAb) responses induced by samRNA vaccine candidates in a South African population.

**Fig. 1:**
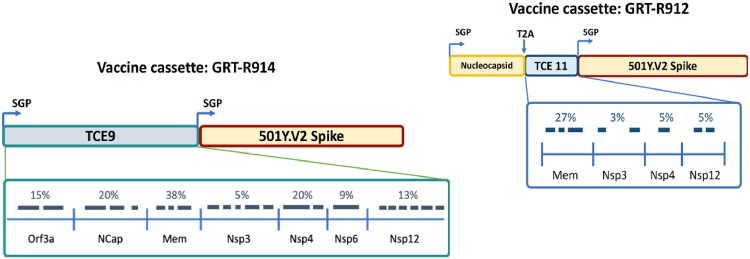
GRT-R914 encoding full-length Beta spike and partial sequences of Nucleocapsid, ORF3a, Membrane, and Non-structural Proteins (NSP) genes to provide T cell epitopes (TCE) outside of Spike GRT-R912 encoding full-length Beta spike and full sequences of Nucleocapsid, ORF3a, Membrane, and Non-structural Proteins (NSP) genes to provide TCE outside of Spike

**Methods:**

This is an ongoing Phase I, open-label, dose-escalation study of samRNA candidates (R914, R912) encoding full-length Spike (S, Beta) and sequences of Nucleocapsid or selected non-S T cell epitopes (TCEs) (Fig.1). R914 (Part A; 3, 10 and 30 mcg) and R912 (Part B; 3 and 10 mcg) were administered as 1 or 2 doses in adults (18-65 years) in two cohorts (Fig.2): SARS-CoV-2 anti-N IgG seronegative or seropositive at baseline. Primary objective is safety and secondary objectives assess (at Vismederi srl) S-specific binding IgG (bAb) and nAbs against Beta and Delta variants of concern (VoC) as well as T cell responses against S and additional TCEs.

**Figure:**
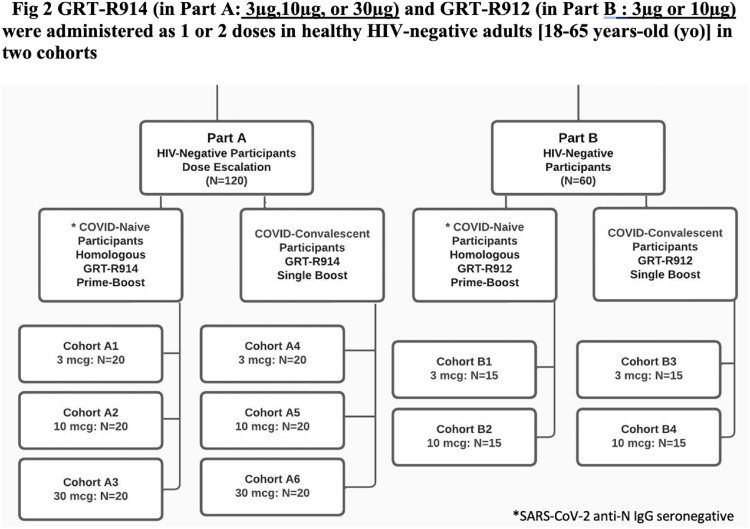

**Results:**

Most reactogenicities were grade 1 or 2 and transient in nature (Fig.3). Ten out of 180 participants reported grade 3 solicited adverse events which resolved within 1-4 days. 99% of participants showed bAbs > 500 ELU/mL at 6 months, while 79% and 70% participants had nAbs > 500 ND_50_ against Beta (included in vaccine), and Delta (cross-reactive), at 6 months in Part A, respectively. Antibody responses were durable up to at least 6 months after R914 and up to at least D57 after R912. The majority of subjects who received GRT-R914 had T cell responses to Non-Structural Protein (NSP) and Nucleocapsid antigens. T cell data from subjects vaccinated with GRT-R912 is pending.

Fig.3

**Figure:**
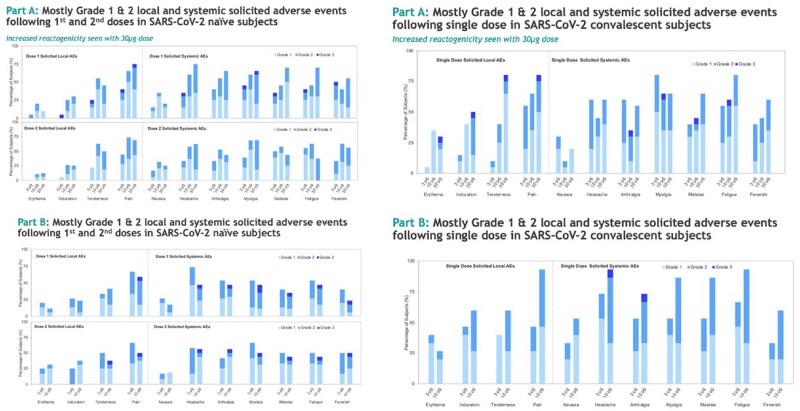

**Conclusion:**

All doses of R914 and R912 were well-tolerated. Both vaccine candidates increased IgG titers against WT as well as nAb titers against selected VoCs. Antibody levels were durable up to at least 6 months after R914 and 57 days after R912 at all dose levels. Humoral immunity against additional VoCs and additional T cell data will be presented. These results showing similar nAb durability are observed with another samRNA vaccine assessed as boost following primary series with ChAdOx1 or mRNA (NCT05148962).

**Disclosures:**

**A. Koen, MD**, Wits Vaccines & Infectious Diseases Analytics (VIDA) Research Unit, South Africa – Johannesburg (South Africa): Employee **E. Mitha, MBChB**, Newtown Clinical Research Centre, South Africa - Newtown (South Africa): Employee **J. Allagappen, MD**, Setshaba Research Centre - Pretoria (South Africa): Employee **A. Nagare, MBBS**, Gritstone bio, Inc.: Employee|Gritstone bio, Inc.: Stocks/Bonds **M. Dhar, MBBS**, Shandukani Research - Johannesburg (South Africa): Employee **M. Marrali, PhD**, Gritstone bio, Inc.: Employee|Gritstone bio, Inc.: Stocks/Bonds **Christine Palmer, PhD**, Gritstone bio, Inc.: Employee|Gritstone bio, Inc.: Stocks/Bonds **Harshni Venkatraman, MS**, Gritstone bio, Inc.: Employee|Gritstone bio, Inc.: Stocks/Bonds **Jason Jaroslavsky, MS**, Gritstone bio, Inc.: Employee|Gritstone bio, Inc.: Stocks/Bonds **JC Kuan, PhD**, Gritstone bio, Inc.: Employee|Gritstone bio, Inc.: Stocks/Bonds **L. Kraemer, PhD**, Gritstone: Employee|Gritstone: Stocks/Bonds **L. Arcebuche, PhD**, Gritstone bio: Employee|Gritstone bio: Stocks/Bonds **L. Hernandez, PhD**, Gritstone bio: Employee|Gritstone bio: Stocks/Bonds **Meghan Hart, ALM**, Gritstone bio, Inc.: Employee|Gritstone bio, Inc.: Stocks/Bonds **Sonia Kounlavouth, BS**, Gritstone bio, Inc.: Employee|Gritstone bio, Inc.: Stocks/Bonds **Pedro Garbes, MD**, Gritstone bio, Inc.: Employee|Gritstone bio, Inc.: Employee|Gritstone bio, Inc.: Stocks/Bonds|Gritstone bio, Inc.: Stocks/Bonds **A. Allen, MBBS, PhD**, Gritstone bio, Inc.: Employee|Gritstone bio, Inc.: Ownership Interest|Gritstone bio, Inc.: Stocks/Bonds **Karin Jooss, PhD**, Gritstone bio: employee|Gritstone bio: Stocks/Bonds **Shabhir A. Mahdi, PhD**, Wits Vaccines & Infectious Diseases Analytics (VIDA) Research Unit, South Africa – Johannesburg (South Africa): Employee

